# Scrotal Abdomen: Case Report and Management Principles

**DOI:** 10.7759/cureus.29113

**Published:** 2022-09-13

**Authors:** Sri Vengadesh Gopal, Selvakumaran Selvaraju, Vivek Sanker, Saravanan Pandian

**Affiliations:** 1 Surgery, Indira Gandhi Medical College and Research Institute, Puducherry, IND

**Keywords:** meshplasty, case report, scrotal, abdomen, inguinal hernia

## Abstract

Hernias extending beyond the midpoint of the inner thigh in the standing position are called giant inguinal hernias or scrotal abdomen. They are rarely seen in common surgical practice. Huge inguinal hernias occur after years of neglect by the patient or in areas that are inaccessible to surgical services. Two cases of giant inguinal hernias which were managed successfully are presented here.

Case 1: 80-year-old male patient presented with left giant scrotal abdomen for the past 12 years. Preoperatively, the pulmonary function test was found to be normal for his age. He was given incentive spirometry for a week. Perioperatively, the sac contained the entire small bowel, sigmoid colon, and omentum with inter bowel loop adhesions. Adhesions were released and it was repaired by hernioplasty with left orchidectomy. In the postoperative period, the patient was put on non-invasive ventilation for two days and then later was weaned off.

Case 2: 42 years male patient presented with right-sided giant inguinoscrotal swelling for the past 15 years. The swelling was extending below midthigh. All the preoperative investigations were normal. Perioperatively, the sac contained omentum and small bowel and it was repaired by right hernioplasty. The postoperative period was uneventful and the patient recovered well.

These are interesting cases of giant inguinal hernias. The occurrence of such potentially dangerous surgical problems is more common in low-to-middle income countries owing to the unavailability of surgical services. The management involves specific measures to prepare the patient adequately preoperatively especially to prevent respiratory complications in the postoperative period.

Giant inguinal hernias can be comfortably managed if the patients are prepared adequately in the preoperative period. Their postoperative period will be uneventful if their pulmonary functions are normal.

## Introduction

Inguinal hernia is the most common surgical condition and hernioplasty is one of the most common operations done in surgical practice. Inguinal hernias can be classified as giant inguinal hernias (GIHs) or scrotal abdomen if they fall below the midpoint of the thigh in the standing position [1], or display an anteroposterior diameter of at least 30 cm or a latero-lateral diameter of about 50 cm [2]. Risk factors leading to giant hernia include chronic obstructive pulmonary disease (COPD), connective tissue disorders, and conditions that may raise intra-abdominal pressure such as obesity and strenuous work. Huge inguinal hernias occur after years of neglect by the patient or in areas that are inaccessible to surgical services [3]. Management of GIH is a challenging one. Many preoperative preparatory methods are mentioned in the literature. Common postoperative complications are respiratory distress and abdominal compartment syndrome. We present two cases of GIH managed in a tertiary care hospital.

## Case presentation

Case report 1 

**Figure 1:**
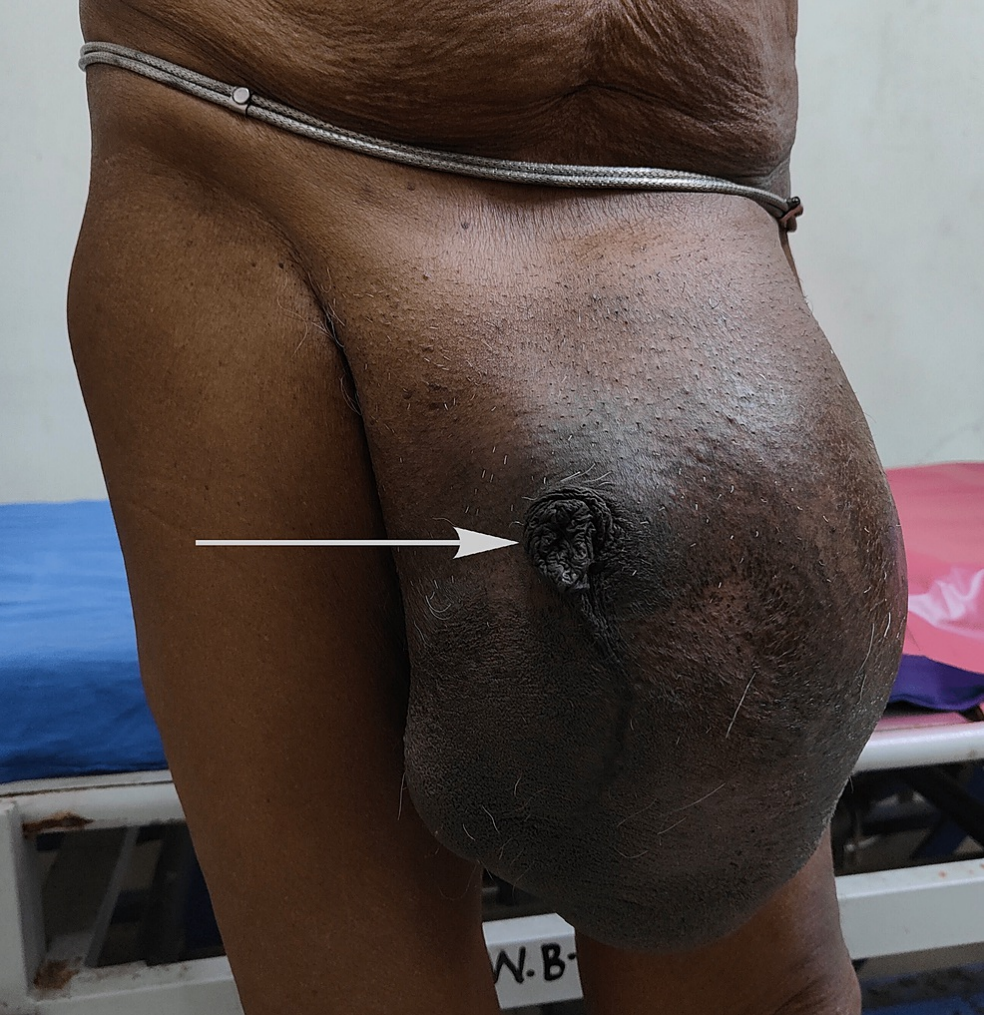
Giant left inguinoscrotal hernia with a buried penis.

An 80 years-old male presented with swelling in the left inguinoscrotal region for the past 12 years with occasional pain inside the swelling. The onset was insidious, progressive, and reached a size of 15 x 15 cm. The inguinoscrotal swelling increased in size and reached the left knee joint (Figure 1).

The GIH was aggravated by straining and coughing and partially reduced in size on lying down. The patient complained of pain at the hernial site on prolonged standing. The patient also had a small direct inguinal hernia on the right side. Penis was buried inside the swelling. His preoperative pulmonary function test (PFT) was normal for his age. Blood investigations, chest X-ray, and electrocardiograph were normal. The patient had systemic hypertension which was well controlled with medications. Preoperatively the patient was admitted to the surgery ward and was given incentive spirometry for a week and then underwent surgery. The patient was put on a fluid diet for two days and also received a low glycerine enema a day before surgery.

**Figure 2:**
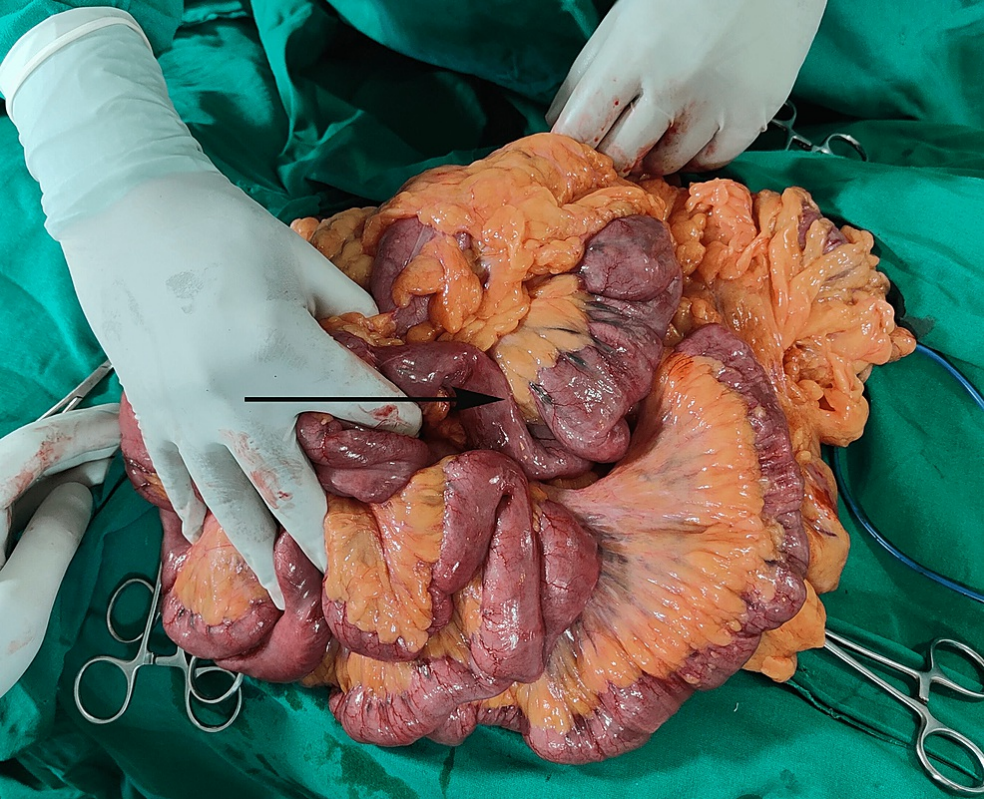
Perioperative picture showing small bowel loops in the hernia sac.

**Figure 3:**
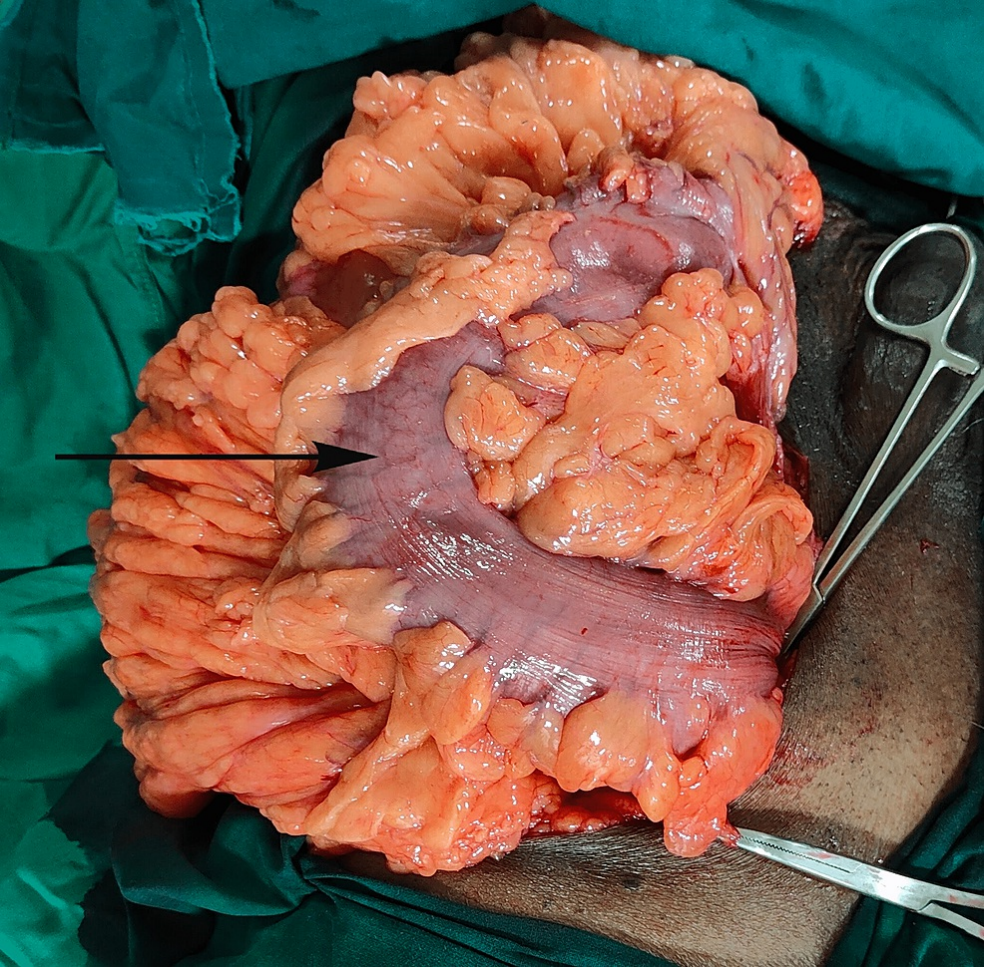
Perioperative picture showing large bowel loops in the hernia sac.

Under epidural anaesthesia, bilateral hernioplasty with left orchidectomy was performed. Findings included a large indirect inguinal hernia extending up to the knee. The sac contained almost the entire small bowel loop, sigmoid colon, and part of the colon was forming the wall of the sac as a sliding hernia (Figure 2 and Figure 3).

The neck of the sac was broad. There were inter bowel loop adhesions in the small bowel loop and also there were adhesions of the small and large bowel with the distal part of the sac. Adhesions were released and the bowel loops were pushed into the abdomen.

**Figure 4:**
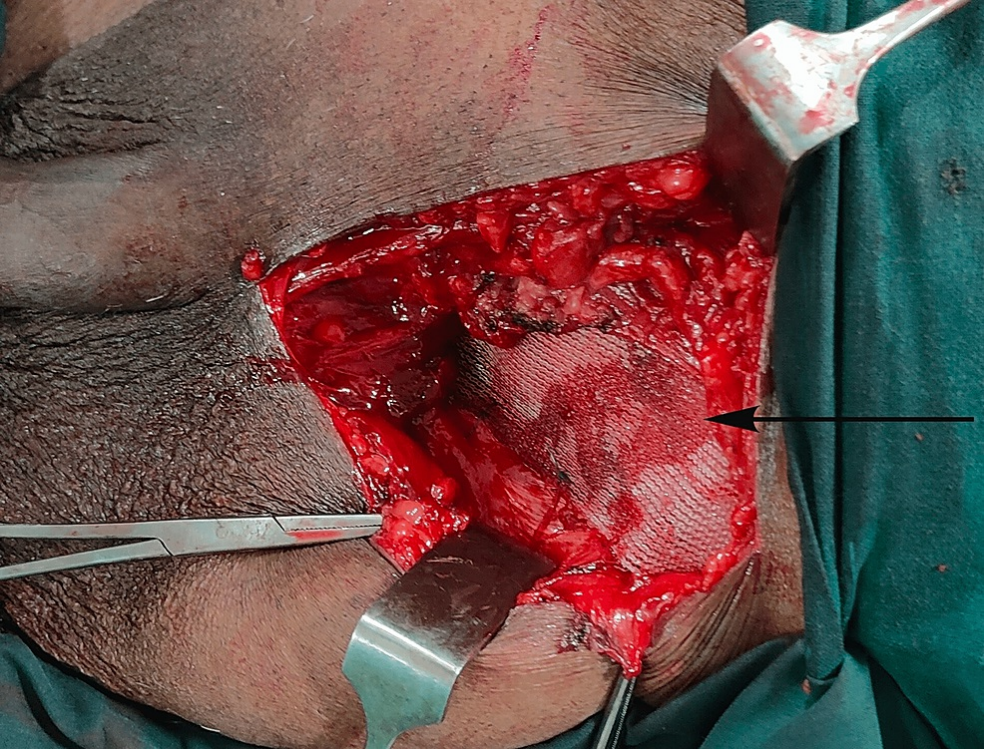
Perioperative picture showing polypropylene mesh fixed over posterior wall.

After the left orchidectomy, the sac was sutured into a deep ring after reducing the contents into the abdominal cavity. The posterior layer was first plicated with 1-0 polypropylene and a polypropylene mesh of size 12 x 5 cm was placed over the posterior wall and fixed using 2-0 polypropylene (Figure 4).

The external oblique layer was closed using 1-0 polyglactin. The corrugated drain was placed in the distal sac in the left hemiscrotum and fixed using 1-0 silk. The subcutaneous layer was closed with 2-0 polyglactin and the skin was closed with a stapler. The scrotal skin was retained and no scrotoplasty was done. In the immediate postoperative period, the area had shrunken to one-fourth of the preoperative size.

Postoperatively, the patient was shifted to the intensive care unit (ICU) and monitored. He was put on oxygen by mask with a flow rate of 10 L/min to achieve an oxygen saturation (SPO_2_) of 95%. Since the patient started desaturating on postoperative day one (POD), he was put on noninvasive ventilation (NIV) for the next two days. Subsequently, NIV was weaned and the oxygen requirement by mask was also reduced. On POD 4, the patient was started on oral fluids and passed flatus on POD 5. He was later shifted from the ICU to the general ward on POD 6. He was off oxygen and maintained SPO_2_ of 98% on room air from POD 6. Aggressive chest physiotherapy was provided to him. He developed a small distal collection in the left scrotum which was aspirated under sterile aseptic precautions. The scrotal skin had shrunken to one-fifth of the preoperative size at discharge and the patient was discharged on POD 10 after removing skin staplers. The patient did well with no evidence of recurrence or complications, and the wound healed well.

Case report 2

**Figure 5:**
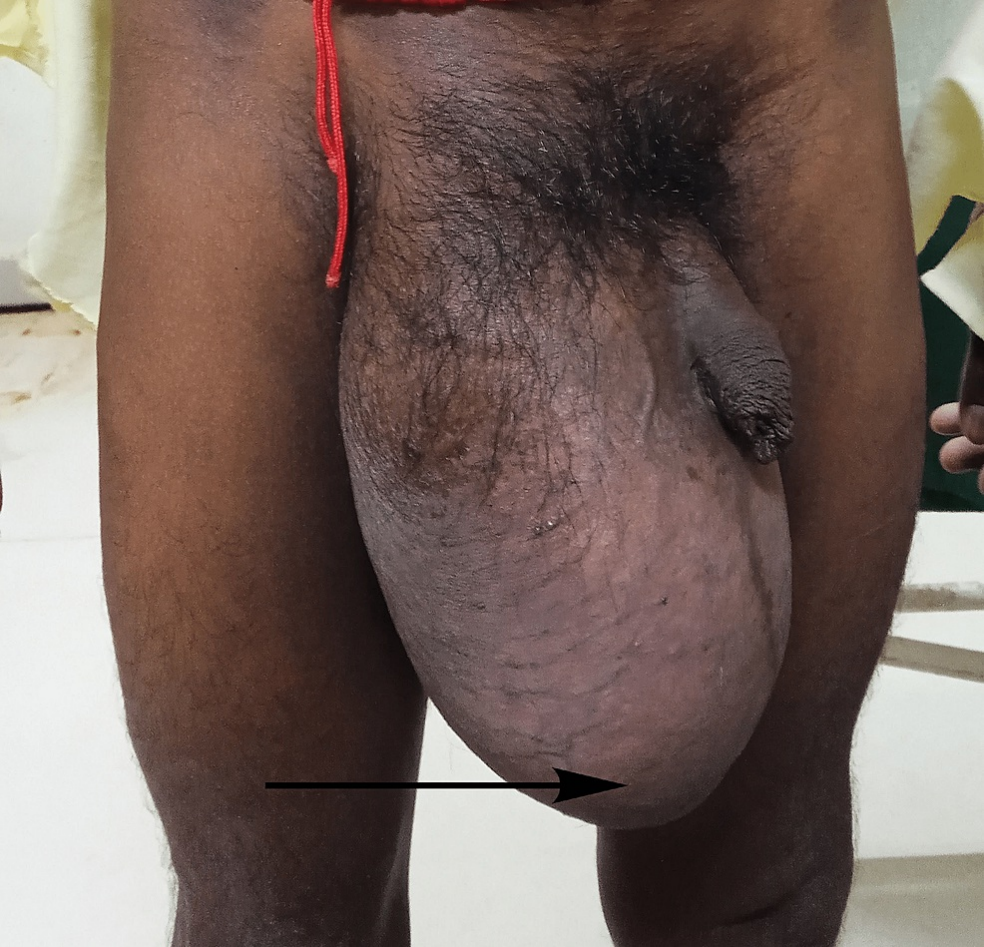
Giant right inguinoscrotal hernia extending beyond mid-thigh.

A 42-year-old male patient presented with swelling in the right inguinal region for the past 15 years. The onset was insidious and progressive and the swelling attained a size of 25 x15 cm. It started from the inguinal region and then descended to the scrotum. The scrotal swelling increased enormously in size and extended beyond the level of the mid-thigh in a standing position (Figure 5).

The swelling increased in size on straining and coughing and completely decreased in size on lying down.

Preoperatively, all routine investigations were done including PFTs, and were found to be normal for his age. He was given incentive spirometry for a week and then underwent surgery. The patient was put on a fluid diet for 2 days and received low glycerine enema a day before the surgery. Under spinal anaesthesia, a right hernioplasty was done. Intraoperative findings included a large indirect inguinal hernia of the right side. The contents within the hernia sac included the majority of the small bowel and greater omentum. The contents of the sac were reduced and the sac was transfixed at the deep ring. A polypropylene mesh of size 12 x 5 cm was placed over the posterior wall and sutured with 2.0 polypropylene. Haemostasis was achieved and the wound was closed in layers. The patient maintained oxygen saturation in room air during the postoperative period and recovered well. He was started on a liquid diet on POD 1. The patient was discharged on POD 8 after the removal of skin sutures.

## Discussion

GIHs are uncommon in today’s surgical practice. The occurrence of such potentially dangerous surgical problems is more common in underdeveloped countries owing to the unavailability of surgical services, inability to afford care, and fear or mistrust of surgical interventions.

Management of GIHs presents several problems. The size itself can interfere with locomotion, the penis can get buried inside the scrotum causing dribbling of urine resulting in excoriations, ulcerations, and secondary infections. It is further aggravated by venous and lymphatic oedema [4]. Other fatal complications include intestinal obstruction, incarceration, and strangulation [5]. The common contents of inguinal hernia are small bowel and omentum, but other structures such as caecum, appendix, bladder, ovaries, and even the entire mesentery and colon have been reported [6,7]. Repair of GIHs can be associated with many complications. The rise in intra-abdominal pressure after reduction pushes up the diaphragm and can cause respiratory failure and pneumonia in addition to wound dehiscence or recurrence. Another problem is that of the large residual scrotal skin [8,9].

Improved prognosis is seen with decreasing the bulk of the contents preoperatively. The use of elemental diets to reduce fecal residues and gastrointestinal secretions is suggested. Another common practice is the resection of the omentum, small bowel, or colon [10,11]. To avoid the development of an abdominal compartment syndrome resulting from a sudden elevation of the intra-abdominal pressure following organ repositioning, the preoperative administration of progressive pneumoperitoneum therapy was suggested [12,13]. Recurrence is common with conventional repairs. Some authors discourage scrotal reconstruction as a precaution so that the contents can be temporarily reverted in case of respiratory failure postoperatively [14].

Older techniques like phrenicectomy, iatrogenic incisional hernia, and musculoskeletal flaps are no longer used [15]. Moreno et al. described the use of artificial pneumoperitoneum preoperatively in ventral hernias [16]. Merret et al. have managed a case of GIH by creating a midline anterior abdominal wall defect and then covering both the hernial and the midline defect by marlex mesh. The mesh was covered by a rotation flap of the redundant inguinoscrotal skin [17].

Mehendale et al. have managed a GIH by debulking contents by performing a right hemicolectomy and small bowel resection and reconstruction of the abdominal wall using marlex mesh and a fascia lata flap [9]. Preperitoneal mesh hernioplasty for such giant hernias has also been described. Considerable mortality and morbidity are present in lower socioeconomic settings because of failure in delivering early interventions for such common surgical cases.

The common reasons for late presentations of such cases include unavailability of surgical services, fear or mistrust of surgical interventions, and inability to afford care [3]. A study in Guatemala demonstrated that most patients with an incarcerated hernia did not seek medical attention because of family obligations and the majority did not have any formal education as well. They concluded that emergent hernias are due to patient-related issues and not due to limitations in the health care system [18]. In our first case, the patient had a history of a road traffic accident 15 years back and sustained a cranial fracture, left clavicle fracture, and hip dislocation and thus neglected the treatment of hernia which lead to the late presentation with a giant scrotal abdominal hernia.

## Conclusions

The management of GIH poses unique challenges. Careful pre-operative preparation and close post-operative monitoring are essential for its successful repair. Patients with good pulmonary function can undergo surgery safely. Scrotoplasty is not usually required since the scrotal skin shrinks very much after the reduction of contents into the peritoneal cavity.

## References

[1] Hodgkinson DJ, Mcllrath DC (1984). Scrotal reconstruction for giant inguinal hernias. Surg Clin North Am.

[2] Cavalli M, Biondi A, Bruni PG, Campanelli G (2015). Giant inguinal hernia: the challenging hug technique. Hernia.

[3] Sturniolo G, Tonante A, Gagliano E (1999). Surgical treatment of the giant inguinal hernia. Hernia.

[4] Lee SE (2012). A case of giant inguinal hernia with intestinal malrotation. Int J Surg Case Rep.

[5] Coetzee E, Price C, Boutall A (2011). Simple repair of a giant inguinoscrotal hernia. Int J Surg Case Rep.

[6] Weitzenfeld MB, Brown BT, Morillo G (1980). Scrotal kidney and ureter: an unusual hernia. J Urol.

[7] Nagendran T (1977). Stomach contained in a giant scrotal hernia. Am Surg.

[8] Valliattu AJ, Kingsnorth AN (2008). Single-stage repair of giant inguinoscrotal hernias using the abdominal wall component separation technique. Hernia.

[9] Mehendal FV, Taams KO, Kingsnorth AN (2000). Repair of a giant inguinoscrotal hernia. Br J Plast Surg.

[10] Serpell JW, Polglase AL, Anstee EJ (1988). Giant inguinal hernia. Aust N Z J Surg.

[11] Kyle SM, Lovie MJ, Dowle CS (1990). Massive inguinal hernia. Br J Hosp Med.

[12] Moreno IG (1947). Chronic eventrations and large hernias; preoperative treatment by progressive pneumoperitomeum; original procedure. Surgery.

[13] Handelsman JC (1959). A technique for increasing abdominal capacity in the repair of massive ventral hernia. Surg Gynecol Obstet.

[14] Vasiliadis K, Knaebel HP, Djakovic N, Nyarangi-Dix J, Schmidt J, Büchler M (2010). Challenging surgical management of a giant inguinoscrotal hernia: report of a case. Surg Today.

[15] Touroff AS (1954). Phrenicectomy as aid to repair of large abdominal hernias. J Am Med Assoc.

[16] Connolly DP, Perri FR (1969). Giant hernias managed by pneumoperitoneum. JAMA.

[17] Merrett ND, Waterworth MW, Green MF (1994). Repair of giant inguinoscrotal inguinal hernia using marlex mesh and scrotal skin flaps. Aust N Z J Surg.

[18] Ochoa-Hernandez A, Timmerman C, Ortiz C, Huertas VL, Huerta S (2020). Emergent groin hernia repair at a county hospital in Guatemala: patient-related issues vs. health care system limitations. Hernia.

